# Influence of the Hydrogen Doping Method on the Atomic Structure, Mechanical Properties and Relaxation Behaviors of Metallic Glasses

**DOI:** 10.3390/ma16041731

**Published:** 2023-02-20

**Authors:** Jiacheng Zhang, Pengfei Gao, Weixu Zhang

**Affiliations:** 1 State Key Laboratory for Strength and Vibration of Mechanical Structures, School of Aerospace Engineering, Xi’an Jiaotong University, Xi’an 710049, China; 2 Northwest Institute of Nuclear Technology, Xi’an 710024, China

**Keywords:** hydrogen doping, metallic glass, molecular dynamics, deformation mechanism, atomic structure, relaxation

## Abstract

The interaction of metallic glasses (MGs) with hydrogen can trigger many interesting physical, chemical and mechanical phenomena. However, atomic-scale understanding is still lacking. In this work, molecular dynamics (MD) simulations are employed to study the atomic structure, mechanical properties and relaxation behaviors of H-doped Ni50Al50 MGs doped by two methods. The properties of H-doped MGs are determined not only by the hydrogen content but also by the doping method. When H atoms are doped into the molten state of samples, H atoms can fully diffuse and interact with metallic atoms, resulting in loose local atomic structures, homogeneous deformation and enhanced β relaxation. In contrast, when H atoms are doped into as-cast MGs, the H content is crucial in affecting the atomic structure and mechanical properties. A small number of H atoms has little influence on the elastic matrix, while the percolation of shear transformation zones (STZs) is hindered by H atoms, resulting in the delay of shear band (SB) formation and an insignificant change in the strength. However, a large number of H atoms can make the elastic matrix loose, leading to the decrease in strength and the transition of the deformation mode from SB to homogeneous deformation. The H effects on the elastic matrix and flow units are also applied to the dynamic relaxation. The deformability of H-doped Ni50Al50 MGs is enhanced by both H-doping methods; however, our results reveal that the mechanisms are different.

## 1. Introduction

Hydrogen is generally considered a kind of point defect in metals and alloys, which can interact with other defects and significantly reduce the ductility of materials even when the H content is quite low. This phenomenon is known as hydrogen embrittlement (HE) [1,2,3,4]. The detrimental effect of H is a long-standing problem for structural materials. Thus, the effect of H on the mechanical properties of metallic materials is always a key issue in the field of scientific research and engineering applications.

Metallic glasses (MGs) are a class of alloys with unique physical, chemical and mechanical properties due to their amorphous structures [5,6,7,8]. There is a large amount of free volume in MGs instead of traditional defects such as dislocations and vacancies. The free volume can be a good placeholder for H atoms [9]. In addition, the complex chemical composition of MGs makes the interaction between H and metallic atoms more complicated. Therefore, most theories about the interaction of H and crystalline materials cannot be applied to MGs directly. Considerable research has been devoted to understanding the effect of H on the mechanical behaviors of MGs [10,11,12,13,14,15]. However, conflicting observations are found in the literature. In earlier studies, it was accepted that H doping can lead to the embrittlement of MGs. HE has been found in multiple MG systems, e.g., Fe- [12], Zr- [16], Ni- [17] and Ti- [18] based MGs. The mechanism of HE in MGs is explained in terms of several different aspects, e.g., the formation of high-pressure bubbles, the excess volume induced by H atoms and the site occupancy of H atoms [19]. In contrast, some studies have reported that H can improve the mechanical properties of MGs, including significant improvement in ductility and enhanced strength [14,20,21,22,23,24]. For example, Dong et al. prepared Zr-based MGs under a gaseous mixture of H_2_/Ar and found that hydrogenation can markedly improve the deformability of MGs without a significant change in strength [21,25]. In their molecular dynamics (MD) simulations, they observed that H atoms cause an increase in the numbers of solid-like atomic clusters and liquid-like clusters. The increase in solid-like atomic clusters makes the framework structure of MGs more stable and thus improves the strength of MGs. The increase in liquid-like atomic clusters can provide more sites for shear transformation zones’ (STZs’) nucleation, which can enhance the ductility of MGs [14,26]. Recently, Tian et al. reported that H-doped Cu-Zr MG, fabricated by charging in a H_2_ atmosphere, displays improved deformability without sacrificing strength [23]. These contradictory phenomena call for more investigations of the effect of H doping on the atomic structures and mechanical behaviors of MGs.

There are many different hydrogen-doping methods in experiments, which may affect the interaction between H and metallic atoms [13,21,23]. Therefore, in this work, we apply two H-doping methods by MD simulations. The details will be mentioned in the computational methods section. The influences of H on the atomic structure, deformation mechanism and relaxation behavior of Ni_50_Al_50_ metallic glass are studied. The partial pair-distribution function $g_{\alpha \beta }\;\left(r\right)$ and Voronoi polyhedral cluster are used to analyze the local atomic structure of MGs. Uniaxial tension tests and molecular dynamics–dynamic mechanical analysis (MD-DMA) tests are applied to study the mechanical behavior and relaxation behavior of MGs. The doping method and H content have crucial roles in the atomic structure, mechanical properties and relaxation behaviors of H-doped MGs. Our simulation results shed light to previous experimental results and advance our understanding of the effects of H on metallic materials.

## 2. Computational Methods

**Figure 1:**
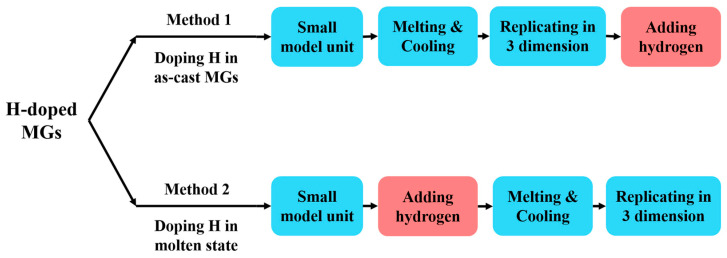
Overview of the two hydrogen-doping methods. The step when H is introduced is highlighted in the red box.

MD simulations are performed using the Large-scale Atomic/Molecular Massively Parallel Simulator (LAMMPS) [27]. The interatomic interactions are described by an embedded atom method (EAM) potential fitted to the Ni-Al-H system [28,29,30]. Two H-doping methods are used to construct H-doped Ni_50_Al_50_ MG samples, as shown in Figure 1. The first method is doping H atoms into as-cast MGs, and the second method is doping H atoms into the molten state of the system. The main difference between the two methods is the sequence to dope H. For method 1, a small model unit (2000 atoms) with periodic boundary conditions (PBCs) along all three independent directions is first equilibrated at 2000 K for 2 ns and then cooled at a quenching rate of $2.5\times 10^{10}$ K/s to 300 K at zero external pressure (NPT ensemble). The large Ni_50_Al_50_ MG sample, containing ~512,000 metallic atoms in a slab with dimensions of $47.5\;\left(x\right)\times 23.7\;\left(y\right)\times 5.9\;\left(z\right){\;\mathrm{nm}}^{3}$, is then constructed by replications of this small cube, annealed for 0.5 ns at 600 K, and finally brought back to 300 K [31,32,33,34]. The H-doped Ni_50_Al_50_ MG is generated by randomly adding H atoms throughout the system, followed by an equilibrium process at 300 K for 200 ps. For method 2, H atoms are randomly added into the system during the liquid stage in the melt–quench process. The H content is denoted by the ratio of H atoms to metallic atoms, H/M. For both doping methods, H-doped MGs with different H contents, defined as the ratio of the number of H atoms to that of metallic atoms, H/M, are prepared.

For uniaxial tension tests, PBCs are imposed along all three dimensions. Tension deformation at a rate of 1 × 10^8^ s^−1^ is applied along the x-direction, and the system temperature is held at 300 K [35]. The pressure in the y- and z-directions is kept at zero using the constant number, pressure, and temperature (NPT) ensemble, allowing for lateral expansion. The deformation processes are analyzed by visualizing the von Mises shear strain [36,37], $\eta_{i}^{Mises}$, calculated with the Open Visualization Tool (OVITO) software [38].

In MD-DMA tests, a structure model containing 16,000 atoms is adopted. PBCs are imposed along all three dimensions. The H content is constructed as H/M = 0.01 and 0.1 by two H-doping methods. A sinusoidal strain of $\varepsilon_{x}\left(t\right)=\varepsilon_{0}\sin\left(2\pi t/\tau_{0}\right)$ is applied along the *x*-direction, where $\varepsilon_{0}$ represents the maximum strain value and $\tau_{0}$ means the period of stress. Here, $\varepsilon_{0}$ is selected as 0.02 and $\tau_{0}$ as 500 ps. All of these MD–DMS tests are carried out in a constant number, volume, and temperature (NVT) ensemble. The corresponding stress-time curves of the samples are fitted by the formula $\sigma_{x}\left(t\right)=\sigma_{c}+\sigma_{0}\sin\;\left(2\pi t/\tau_{0}+\delta \right)$, where $\sigma_{c}$, $\sigma_{0}$ and δ are fitted parameters. The storage modulus E′ and loss modulus E″ are calculated as $E^{\prime }=\left(\sigma_{0}/\varepsilon_{0}\right)\;\cos\delta$ and $E^{\prime \prime }=\left(\sigma_{0}/\varepsilon_{0}\right)\;\sin\delta$, respectively [39,40,41].

## 3. Results and Discussion

### 3.1. Short-Range Order

**Figure 2:**
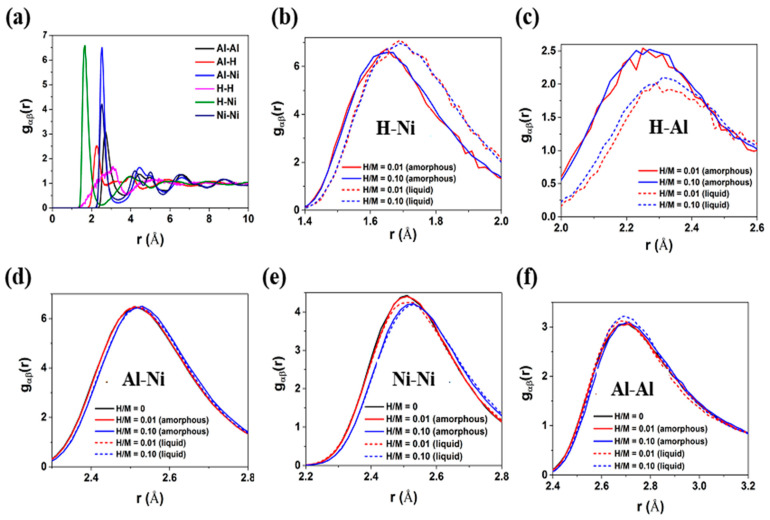
(**a**) The typical $g_{\alpha \beta }\;\left(r\right)$ of H-doped Ni_50_Al_50_ MGs; (**b**,**c**) the first peak of H-Ni and H-Al pairs; (**d**–**f**) the first peak of Al-Ni, Ni-Ni and Al-Al pairs. The solid line is the H-free MG or samples prepared via method 1, and the dashed line is samples prepared via method 2.

The $g_{\alpha \beta }\;\left(r\right)$ is calculated to investigate the effects of doping methods and H content on the short-range order of Ni_50_Al_50_ MGs [42,43], which is defined as $g_{\alpha \beta }\left(r\right)=\frac{D}{N_{\alpha }N_{\beta }}\langle \overset{N_{\alpha }}{\underset{i=1}{\sum }}\frac{n_{i\beta }\left(r\right)}{4\pi r^{2}\Delta r}\rangle$, where $N_{\alpha }$ and $N_{\beta }$ represent the number of α and β atoms, respectively. D denotes the volume of the sample cell, and $n_{i\beta }\;\left(r\right)$ is the number of β atoms that can be found in the shell from r to r+Δr centered on the *i*th α atom. Figure 2a shows the $g_{\alpha \beta }\;\left(r\right)$ of H-doped MGs. The first peak position refers to the first-nearest-neighbor distance, and the peak intensity indicates the order degree of atomic-pair bonding. The first peak position of H-Ni (D_H-Ni_) is approximately 1.65 Å, and that of H-Al (D_H-Al_) is approximately 2.25 Å. This indicates that the distance of H-Ni pairs is shorter than that of H-Al pairs. Considering the radii of ground state atoms, i.e., R_Ni_ = 1.49 Å, R_Al_ = 1.18 Å and R_H_ = 0.53 Å, it is seen that the first peak position of the H–Al pair is much larger than the sum of their atomic radii, i.e., D_H-Al_ > R_H_ + R_Al_ = 1.71 Å. This result highlights that H atoms prefer to bond with Ni atoms instead of Al atoms at the first-nearest-neighbor distance. To further characterize the chemical affinity between atomic pairs, the mixing enthalpy $\Delta H_{\alpha \beta }$ of atomic pairs is calculated based on Miedema’s model [42,44,45]: $\Delta H_{HAl}=-8\;\mathrm{kJ}/\mathrm{mol}$, $\Delta H_{HNi}=-23\;\mathrm{kJ}/\mathrm{mol}$ and $\Delta H_{AlNi}=-22\;\mathrm{kJ}/\mathrm{mol}$. These values are consistent with the results of Inoue et al.’s study [44]. $\Delta H_{HNi}$ is the largest negative value, which indicates that H atoms prefer to bond with Ni atoms. This result is in good agreement with the above-mentioned $g_{\alpha \beta }\;\left(r\right)$ results.

Figure 2b,c show the first peak in $g_{\alpha \beta \;}\left(r\right)$ of H-metal atomic pairs, i.e., H-Ni and H-Al pairs for H-doped MGs with different H contents and H-doping methods. Compared with H-doped Ni_50_Al_50_ MGs prepared via method 1, samples prepared via method 2 have larger D_H-Ni_ (~1.69 Å) and D_H-Al_ (~2.31 Å) and a higher peak intensity of the H-Ni pair and a lower peak intensity of the H-Al pair, indicating that H-Ni pairs are more orderly in these samples. The first peak position and the peak intensity of the H-Ni and H-Al pairs are obviously affected by the H-doping method. For samples prepared with the same doping method, the H content has less effect on the first-nearest-neighbor distribution of H-Ni and H-Al pairs. It can be inferred that the chemical affinity between atomic pairs plays a leading role in the distribution of H atoms in MGs. When H atoms are doped into as-cast Ni_50_Al_50_ MGs, the position of metallic atoms is relatively stable, and H atoms are distributed in the free volume between these metallic atoms. The diffusion of H atoms is restricted, and these H atoms prefer to interact with the nearest Ni atoms, forming metastable H-Ni pairs. When H atoms are doped into the liquid state of the Ni_50_Al_50_ system, H atoms can sufficiently diffuse and interact with Ni atoms during the melting and cooling processes, leading to the generation of stable H-Ni pairs and loose H-Al pairs.

Figure 2d–f show the first peak in $g_{\alpha \beta }\;\left(r\right)$ of metal–metal atomic pairs, i.e., Al-Ni, Ni-Ni and Al-Al pairs for H-doped MGs with different H contents and H-doping methods. The $g_{\alpha \beta }\;\left(r\right)$ of H-free MGs is also plotted for comparison. In contrast to H-metal atomic pairs, the first-nearest-neighbor distribution of metal–metal atomic pairs is influenced by the H content instead of the H-doping method. When the H content is low, H atoms have a marginal impact on metal–metal pairs. With the increase in H atoms, both D_Al-Ni_ and D_Ni-Ni_ have a slight increase, indicating the loose packing of the atomic structure in MGs. Al-Al pairs are less affected because the interaction between H and Al atoms is weak.

**Figure 3:**
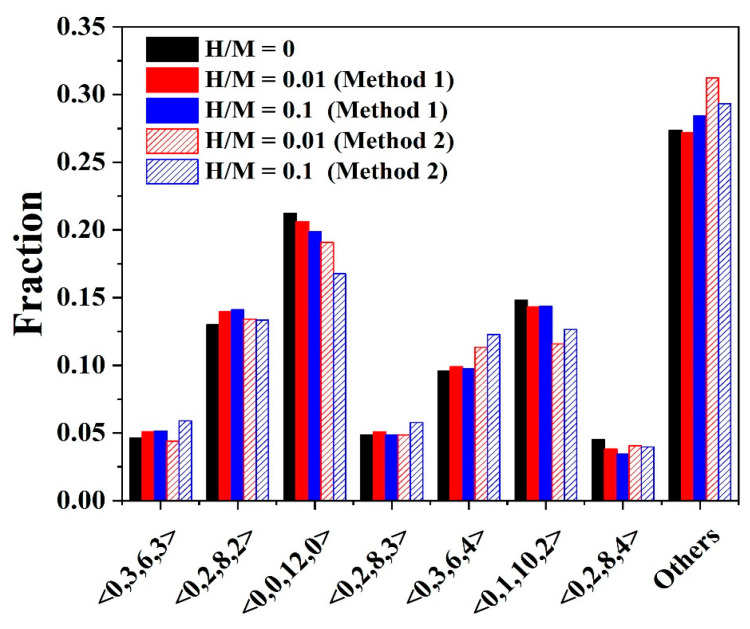
Proportion of main Ni-center atomic clusters in H-free and H-doped Ni_50_Al_50_ MGs.

To further examine the change in atomic structure in Ni_50_Al_50_ MGs after doping with H atoms, based on the random dense packing model, Voronoi polyhedral cluster analysis is employed [46]. Voronoi polyhedra indicate the 3D atomic configuration between the centered and surrounding atoms. The Voronoi index <n_3_, n_4_, n_5_, n_6_> is used to designate and differentiate the type of coordination polyhedron, where n_i_ denotes the number of i-edged faces of the Voronoi polyhedron. Generally, icosahedra polyhedra with a high fraction of fivefold, e.g., <0,0,12,0> and <0,1,10,2>, lead to high-density atomic packing and contribute to the elastic matrix of MGs. Clusters with a low fraction of fivefold are usually loosely packed and have large volumes, and hence easily participate in shear transformation which is partly responsible for the flow units in MGs [47]. Based on the Voronoi analysis by OVITO [38], we have calculated the atomic volume of the Ni-centered clusters in H-free Ni_50_Al_50_ MG to characterize the compactness of Voronoi polyhedra. The atomic volume is defined as the volume of the Voronoi cell of the atom. The average atomic volume of the Ni atoms with a “solid-like” <0,0,12,0> cluster is 11.874 Å^3^, while the value of Ni atoms with a <0,3,6,4> cluster is 12.495 Å^3^. Note that the atomic volume of <0,3,6,4> polyhedra is larger than that of <0,0,12,0> polyhedra. Therefore, it can be considered that <0,0,12,0> clusters are more closely packed than <0,3,6,4> clusters. The fractions of the several most populous Voronoi polyhedra centered around Ni atoms in H-free and H-doped MGs are shown in Figure 3. ‘Other polyhedra’ refers to the group of low-fraction Voronoi polyhedral clusters. Two “solid-like” clusters, <0,0,12,0> and <0,1,10,2>, are the most significant Ni-centered clusters in H-free Ni_50_Al_50_ MG. The fractions of these two clusters are 0.212 and 0.206, respectively. When H atoms are doped into MGs, these two “solid-like” clusters decrease as the number of H atoms increases, especially in samples prepared via method 2. Meanwhile, the loosely packed polyhedra such as <0,3,6,4> and “other” clusters have an obvious increase. This result indicates that H atoms loosen the elastic matrix of Ni_50_Al_50_ MGs. Due to the full interaction of H and metallic atoms, the atomic structure of H-doped Ni_50_Al_50_ MGs prepared via method 2 is more affected by H atoms than that of samples prepared via method 1.

### 3.2. Mechanical Properties

#### 3.2.1. H-Doped MGs Prepared via Method 1

**Figure 4:**
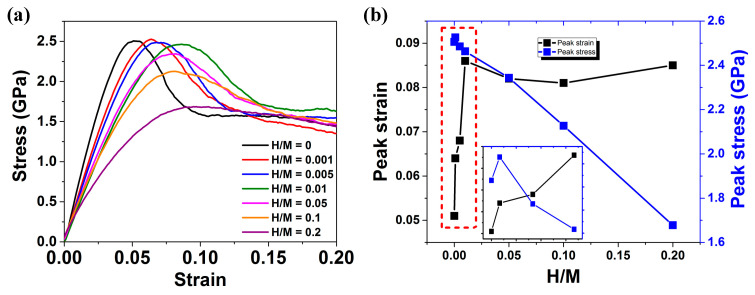
(**a**) Tensile stress–strain curves of H-free and H-doped Ni_50_Al_50_ MGs prepared via method 1. (**b**) The peak strain and peak stress as a function of H content.

Figure 4a depicts the tensile stress-strain curves of H-free and H-doped Ni_50_Al_50_ MGs prepared via method 1. Figure 4b shows the peak stress and the peak strain as a function of H content. The curve of H-free Ni_50_Al_50_ MG shows linear elastic behavior in the early stage, followed by nonlinear behavior before reaching the peak stress. After reaching the peak stress at a strain of ~5%, a stress drop occurs, which corresponds to a rapid localization of the plastic strain into a single dominant shear band (SB). Then, the stress-strain curve becomes flat upon subsequent loading, corresponding to SB sliding. This observation is consistent with previous simulation results of other MGs, e.g., Cu-Zr [48] and Fe-P MGs [49]. When the H content is low (H/M < 0.01), the stress-strain curves of H-doped and H-free MGs exhibit the same three stages: elastic regime, stress drop and flat flow. The peak stress of H-doped MGs experiences little change, while the peak strain increases markedly from ~5% for H-free MG to ~8.5% for H-doped MG with H/M = 0.01. When the H content is high (H/M > 0.05), the peak strain only changes slightly, while the peak stress decreases linearly with increasing H content from ~2.51 GPa for H-free MG to ~1.68 GPa for H-doped MG with H/M = 0.2. Especially for MG with H/M = 0.2, the stress drop disappears. Our results in Figure 4 highlight that when H is doped into as-cast MGs, H-doped MGs exhibit a dramatic failure mode transition from localized shear banding to super ductile behavior with increasing H content [31].

**Figure 5:**
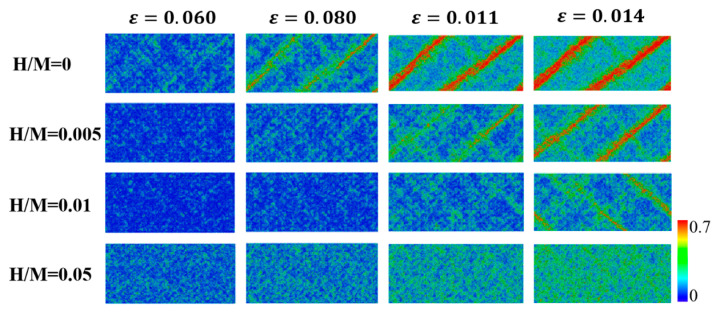
A sequence of snapshots capturing the atomic deformation processes for H-free and H-doped Ni_50_Al_50_ MGs prepared via method 1. The colors indicate the atomic shear strain. The change in the deformation mode from highly localized shear banding to fully homogeneous plastic flow is clearly observed.

To understand the above-mentioned transition in the deformation mechanism, we study the atomic deformation processes by examining the atomic local shear strain $\eta_{i}^{Mises}$ with respect to the relaxed samples prior to tensile loading [37,50]. Figure 5 shows a sequence of snapshots that capture the deformation process for H-free and H-doped Ni_50_Al_50_ MGs prepared via method 1. The regions with relatively large $\eta_{i}^{Mises}$ imply that they have undergone large localized shear strain and host a high density of STZs [51]. For the H-free sample, after the elastic stage, STZs begin to nucleate and quickly form a single dominant SB at a strain of ~0.08. The SB propagation follows the direction of maximum shear stress, which is ~45° relative to the loading axis. These results are consistent with previous MD simulation results [34]. For MGs with low H contents of H/M = 0.005 and H/M = 0.01, it is observed that the formation processes of STZs and SBs are delayed. With increasing H content, the STZ and SB retardation becomes significant, indicating enhanced deformability. When the H content is further increased, e.g., H/M = 0.05, the STZs distribution tends to be well spread through the entire specimen. There is no evidence of a single dominant SB even at very large strain of 0.14. Our results are in good agreement with the simulation results of Luo et al. [14]. Based on the results of Figure 4 and Figure 5, H-doped MGs with high H content exhibit homogeneous plastic flow with strength reduction.

**Figure 6:**
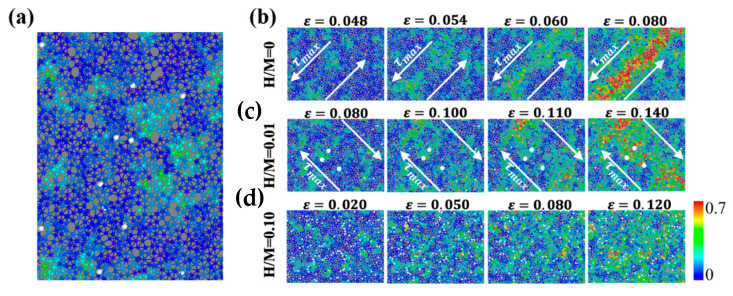
(**a**) The positional relationship between H atoms and STZ nucleation, H/M = 0.01 (method 1) at ε = 0.08. (**b**–**d**) Percolation path of STZs during deformation for samples with different H contents. The white atoms are H atoms, and the other atoms are colored by their $\eta_{i}^{Mises}$.

The results in Figure 4 and Figure 5 raise an important question: what is the underlying mechanism of this H-content-dependent transition? Further analysis of the interaction between H atoms and STZs is performed. Figure 6a depicts the slice of MG with a low H content at a strain of 0.08 to investigate the distribution of H atoms and incipient STZs. The slice thickness is taken as 3 Å, which is equivalent to the nearest-neighbor atomic distance in Ni_50_Al_50_ MGs. The white atoms are H atoms, and metallic atoms are colored by their $\eta_{i}^{Mises}$. H atoms are mainly distributed at the margin of STZs or areas with samll $\eta_{i}^{Mises}$. There is no evidence of H atoms in the central area of STZs. This indicates that a small number of H atoms do not contribute to the formation of STZs. It is possible that the formation of metastable H-Ni pairs inhibits the motion of atoms. Figure 6b present slices of H-free and H-doped Ni_50_Al_50_ MGs prepared via method 1 during deformation. For the H-free sample, as shown in Figure 6b, STZs begin to nucleate near the yield strain. With the increase in deformation, some STZs percolate and interact with each other, promptly following the direction of the maximum shear stress $\tau_{max}$ [51], and a distinct SB is formed at a strain of ~0.08. Three H atoms are highlighted to emphasize the interaction between H atoms and STZs. H atoms are originally distributed around the margin of STZs at a strain of ~0.08. When the tensile loading is further increased, the percolation expansion of STZs is obviously hindered by these H atoms instead of directly following the direction of $\tau_{max}$. The area surrounding the H atoms maintains small deformation until a single SB is formed. For an MG with a high H/M ratio of 0.1, as shown in Figure 6d, STZs are prone to nucleate at a small strain of ~0.02. More STZ events nucleate and uniformly disperse through the whole sample, which is in striking contrast to the single SB observed in H-free MG and H-doped MGs with low H contents. This change indicates that the elastic matrix is loosened when a large number of H atoms are doped, as discussed in Figure 2 and Figure 3.

**Figure 7:**
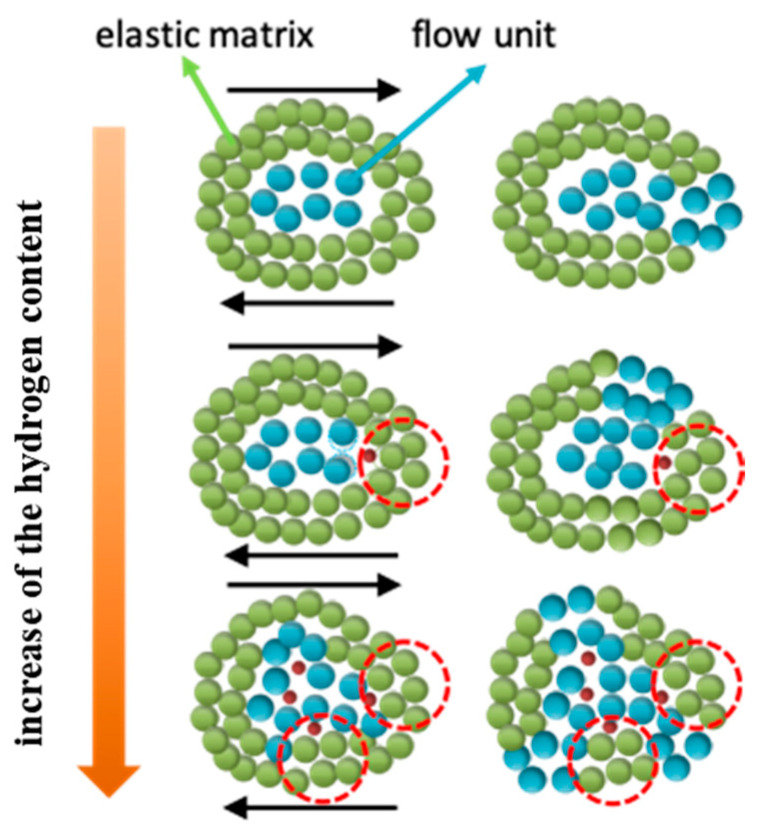
Schematic diagram of the H loosing effect on the elastic matrix and the H pinning effect on STZs. Green atoms represent the elastic matrix, blue atoms represent flow units, red atoms are H atoms and red dotted lines represent clusters that remain relatively stable during the deformation process. The black arrows represent the direction of maximum shear stress.

From the above analysis, it can be concluded that the SB formation process is influenced by the percolation behavior of STZs involving H atoms, and the strength is affected by the number of solid-like clusters in H-doped MGs. Our results on H-doped MGs prepared via method 1 are summarized in Figure 7. The H effect strongly depends on the H content. For H-free MGs, STZs nucleate at flow units that are loose packing clusters wrapped by the backbone-like elastic matrix. Under external loading, STZs normally percolate along the direction of maximum shear stress, which results in the formation of a dominant SB. After adding a small number of H atoms, the influence of H on the elastic matrix of MG is insignificant; hence, the change in strength is not obvious. However, the metastable Ni-H pairs hinder STZ percolation, as observed in Figure 6c, and thus, STZs preferentially percolate along the direction without H atoms. If the hindered direction is exactly the direction of maximum shear stress, the H effect on STZs may impede the accumulation of adjacent STZs and cause a delay in SB formation. This phenomenon is identified as the H pinning effect on STZs. However, with increasing H content, the influence of H atoms on the elastic matrix cannot be ignored. Therefore, the elastic matrix becomes loose, resulting in a decrease in strength. The loose atomic structure benefits the formation of more STZs, and the percolation direction of STZs is less affected by the direction of maximum shear stress. There is no single SB formation due to the homogeneous dispersion of more STZs. The H effect on the atomic structure of MGs is identified as the H loosing effect on the elastic matrix.

#### 3.2.2. H-Doped MGs Prepared by Method 2

**Figure 8:**
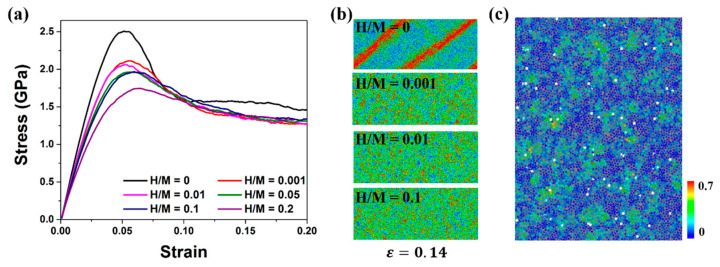
(**a**) Stress-strain curve of H-free and H-doped Ni_50_Al_50_ MGs prepared via method 2 under uniaxial tensile load. (**b**) Deformation diagram of samples with different H contents at ε = 0.14. (**c**) The positional relationship between H atoms and STZs in Ni_50_Al_50_ MG with H content of H/M = 0.01, ε = 0.048; white atoms are H atoms, and other atoms are colored according to their $\eta_{i}^{Mises}$.

Figure 8a shows the tensile stress-strain curves of H-free and H-doped Ni_50_Al_50_ MGs prepared via method 2. The corresponding deformation snapshots of samples with different H contents at a strain of 0.14 are displayed in Figure 8b. The mechanical properties of H-doped Ni_50_Al_50_ MGs prepared via method 2 are significantly different from those of the samples prepared via method 1. With increasing H content, the peak stress decreases, while the peak strain remains almost unchanged at ~0.05. Even when the H content is fairly low (H/M = 0.001), the strength decreases significantly compared with H-free MG. All H-doped MGs undergo homogeneous plastic deformation without a single SB formation. On the basis of Figure 2 and Figure 3, the atomic structure in H-doped MGs prepared via method 2 is looser than that in H-free MGs. Previous studies have shown that the loose atomic structure, especially the decrease in stable clusters such as <0,0,12,0> and <0,1,10,2>, can improve the ductility of MGs, accompanied by a decrease in strength. Here, our results exhibit the same trend. Figure 8c depicts a slice of MG with an H content of H/M = 0.01 at ε = 0.048. The looser atomic clusters provide more fertile sites for STZ nucleation. H atoms disperse randomly in the whole sample, which is in striking contrast to the H distribution in method 1, mainly in regions with small $\eta_{i}^{Mises}$ or the margin of STZs, as shown in Figure 6a. There is no obvious dependence of the H atom position on STZ nucleation sites. The H pinning effect on STZs is no longer valid in samples prepared via method 2.

### 3.3. Relaxation Behaviors

The β relaxation, corresponding to rearrangements of the local atomic structure in glass, is an intrinsic and universal feature of MGs. Previous studies have found that β relaxation and STZs have semblable structural origins and activation energies [52]. Therefore, investigations of dynamic relaxation are significant for understanding the flow behavior, flow units, deformations and mechanical properties of MGs.

**Figure 9:**
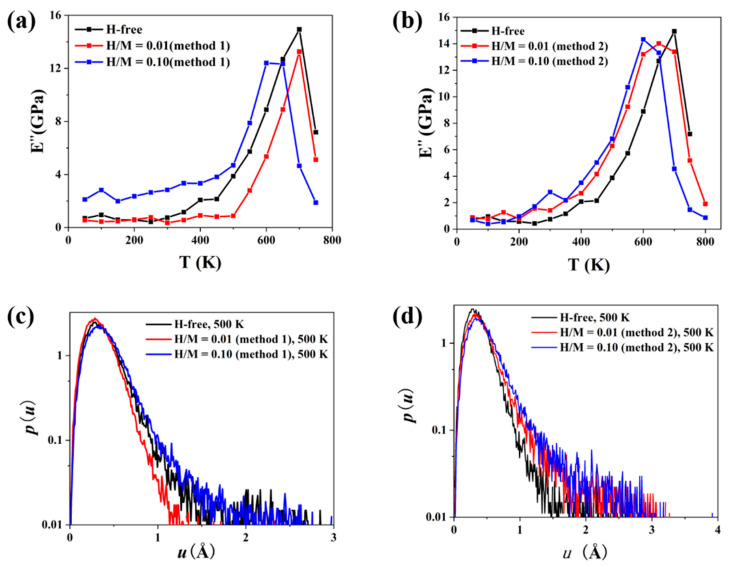
(**a**,**b**) are the temperature spectra of the loss modulus of the glassy hydrogenation and liquid hydrogenation samples, respectively. (**c**,**d**) are the probability density distributions of metal atom displacement after cyclic loading at 500 K.

Figure 9a,b are the temperature spectra of the loss modulus *E*″ of H-doped MGs prepared using the two methods. The spectrum of H-free MGs is also plotted for comparison. All spectra have a main peak at high temperature, corresponding to the transition of samples from the glass state to supercooled liquid state, known as α relaxation [40,52]. For H-doped MGs prepared via method 1, as shown in Figure 9a, *E*″ is strongly dependent on the H content. For an MG with a low H content of H/M = 0.01, *E*″ is lower than that of the H-free sample. This trend is consistent with recent experimental results [14]. For an MG with a high H content of H/M = 0.1, *E*″ is significantly higher than that of the H-free sample. This indicates that a small number of H atoms can suppress the β relaxation of MGs, while a large number of H atoms promotes the β relaxation of MGs. For H-doped MGs prepared via method 2 shown in Figure 9b, the *E*″ for low and high H contents are higher than that of H-free MGs, which is insensitive to the H content.

Atoms in MGs have kinetic inhomogeneity during the relaxation process, which can be characterized by the probability density distribution function p (u) of atom displacement [39]. p (u) is defined by the formula [P (u+Δu)−P (u)]/Δu, where u denotes the atom displacement, P (u) represents the cumulative distribution quantifying the probability of finding that the atom displacement is less than u, and Δu is set to 0.05 Å. The calculated p (u) for H-doped MGs prepared using two methods at 500 K is shown in Figure 9c,d, respectively. The selected 500 K is below the Tg (glass transition temperature) of XX K. Figure 9c shows that the motion of metallic atoms is restrained by low H content but is promoted by high H content in H-doped MGs prepared via method 1. In addition, Figure 9d shows that the movement of metallic atoms is enhanced by H atoms in H-doped MGs prepared via method 2. This is the kinetic origin of the relaxation behavior shown in Figure 9a,d.

**Figure 10:**
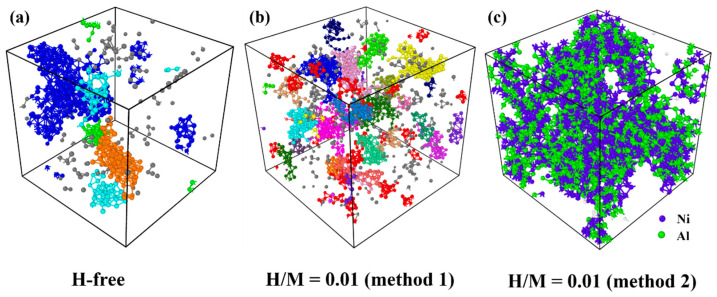
The distribution of flow units in MD-DMA tests of H-free and H-doped Ni_50_Al_50_ MGs at 400 K. In (**a**,**b**), interconnected clusters are colored in the same color. In (**c**), blue atoms are Ni, and green atoms are Al.

To further investigate the influence of H on the flow units in MGs, the activated atomic clusters during MD-DMA tests are analyzed in Figure 10. The metallic atoms with $\eta_{i}^{Mises}>0.2$ are identified as activated atoms with obvious rearrangement behavior. Interconnected clusters are colored in the same color in Figure 10a,b. As shown in Figure 10a, there are a few activated atomic clusters in the H-free MG. The largest cluster contains ~300 atoms. For an H-doped MG prepared via method 1 shown in Figure 10b, the number of activated atomic clusters increases compared to an H-free MG, indicating that more flow units are activated with the introduction of H atoms. However, each cluster contains fewer atoms. The largest cluster contains ~130 atoms. This implies that the interactions among activated atomic clusters weaken, resulting in the percolation of the activated atomic clusters slowing down. Therefore, the H pinning effect is also applied to flow units during dynamic relaxation. As shown in Figure 10c, a considerable number of small atomic clusters undergo structural rearrangement in H-doped MGs prepared via method 2, leading to the homogeneous distribution of activated atomic clusters. This result indicates that the atomic structure is relatively loose and can trigger more flow units for enhanced β relaxation. This is consistent with the abovementioned conclusions that a distinctly loose atomic structure and homogeneous plastic deformation are found in H-doped MGs prepared via method 2.

## 4. Conclusions

The interaction of H atoms with metals and alloys is unavoidable during the service condition. However, experimental studies have reported conflicting results regarding the effect of H on the mechanical properties of MGs. Despite being of the utmost importance, an understanding of the effect of H on the atomic structure and mechanical properties is still lacking. In this work, we perform MD simulations to study the influence of H on the atomic structure, mechanical properties and relaxation behaviors of Ni_50_Al_50_ MGs using two H-doping methods. H atoms are found to bond with Ni atoms. The local atomic structure becomes loose with the introduction of H atoms. For H-doped MGs prepared by doping H into the molten Ni_50_Al_50_ system, H atoms sufficiently diffuse in the whole sample, making the atomic structure loose. This promotes the nucleation and percolation of STZs and enhances the relaxation behavior in MGs. Homogeneous deformation is observed, accompanied by strength reduction. For H-doped MGs prepared by doping H atoms into as-cast Ni_50_Al_50_ MG, the H content is a key factor that influences the atomic structure and mechanical properties. The H pinning effect on STZs and H loosing effect on the elastic matrix are found during uniaxial tensile loading. When the H content is low, the strength experiences little change due to the negligible H loosing effect on the elastic matrix, while the H pinning effect on STZs can lead to the delay of SB formation. When the H content is high, the H loosing effect on the elastic matrix becomes significant, leading to an obvious strength reduction accompanied by homogeneous plastic deformation. The H pinning effect is also applied to the percolation of flow units during dynamic relaxation. This work provides an atomic-scale understanding of the atomic structure, deformation mechanism and relaxation behavior of H-doped MGs and guidelines for the design of ductile MGs.

## Data Availability

The data used to support the findings of this study are available from the corresponding author upon request.
